# The Care Dialog: the “ethics of care” approach and its importance for clinical ethics consultation

**DOI:** 10.1007/s11019-017-9784-z

**Published:** 2017-07-03

**Authors:** Patrick Schuchter, Andreas Heller

**Affiliations:** Institute of Palliative Care and Organisational Ethics, IFF Faculty of Interdisciplinary Studies, Alpen-Adria Universität Klagenfurt | Wien | Graz, Schottenfeldgasse 29/4, 1070 Vienna, Austria

**Keywords:** Care, Ethics, Dialog, Ethics Consultation, Care of the self, Health care ethics, Moral case deliberation, Narrative ethics, Socratic Dialogue, Advance Care Planning, Art of living, Organizational ethics

## Abstract

Ethics consultation in institutions of the healthcare system has been given a standard form based on three pillars: education, the development of guidelines and concrete ethics consultation in case conferences. The spread of ethics committees, which perform these tasks on an organizational level, is a remarkable historic achievement. At the same time it cannot be denied that modern ethics consultation neglects relevant aspects of care ethics approaches. In our essay we present an “ethics of care” approach as well as an empirical pilot project—“Ethics from the bottom up”—which organizes ethics consultation based on this focus. Findings and philosophy of the project will be discussed as far as relevant for ethics consultation in the healthcare system.

## Background

Ethics consultation was originally developed in hospitals. Clinical ethics committees were established, instituting an almost standard organizational form based on three pillars: training in ethical issues, the development of guidelines and the facilitation of ethical case conferences on the ward (cf. Steinkamp and Gordijn 2010; Post et al. 2007; Hester and Schonfeld 2012; Academy for Ethics in Medicine 2010; Schochow et al. 2015). According to Kettner (2008), the prospective case deliberation is the core activity of clinical ethics consultation services and aims at facilitating decision-making when there is uncertainty or a need for clarification in the treatment process. As the Nijmegen method is one of the better known models, we refer to the Steinkamp and Gordijn definition and conversational structure as an example for many comparable models at this point.

According to Steinkamp and Gordijn (2003, 2010), the ethical case deliberation on the ward is an attempt to reach the best ethical decision through a moderated discussion with a multidisciplinary team.

This is accomplished in 4 steps:

1. Definition of the ethical problem
2. Analysis of the medical, nursing, social, ideological and organizational facts
3. Assessment and development of arguments from the viewpoint of ethical norms. (This is the point where the four principles of bioethics according to Beauchamp/Childress—respect for autonomy, nonmaleficence, beneficence, justice—usually come into play.)
4. Decision-making, including a summary of the most important reasons for the decision

Therefore, we talk about

1. A multidisciplinary team, i.e. professional co-workers
2. A situation requiring decision-making (a moral dilemma or conflict)
3. An “analysis” of “facts”, first medical, then nursing etc., and finally
4. Arguments and norms

In terms of organizational theory—referring to constitutional conditions or “input rules” (cf. May 2010, 93 et seq.)—the (prospective) case discussion is in fact an emergency meeting summoned for a specific reason. The output of the consultation refers to a single decision at the end or during treatment routines.

Within a certain range of variations (and without denying exceptions and forms of moral case deliberation which include elements of, for example, the Socratic or Hermeneutic Dialog), it can nevertheless be said in general that these are the main characteristics of ethics consultation within the paradigm of the clinical standard model: moral dilemmas as subject of deliberation, orientation along normative principles, treatment decisions as the objective of ethics consultation, and organization of the deliberation as a special communication in case of ethical emergency. Without denying the utility and merits of the standard model, it can be argued, however, that conceiving ethics in this way also means reducing ethics and obscuring possible issues and deliberation processes, which should also be perceived as ethical issues and ethical deliberations.

In this article, we would like to reconsider this paradigm on some points which, from our point of view, are less self-evident than it might seem, and should therefore be—and partly already are—subjected to further discussion. Preliminarily, we will take a first look at main aspects of these points at issue.

### Focus on moral dilemmas

In a humorous way Kwame Anthony Appiah pointed at a problematic and narrowing focus in current ethics. If you follow its main lines, you can get the impression that the main problem of moral life consists in solving dilemmas. He therefore talks about a “dilemma–dilemma” in modern ethics (Appiah 2009, 198 et seq.). What Appiah rather applied to the dilemma construction in ethical theory development is even more valid in the applied and practiced form of ethics in the healthcare system. In the currently predominant organizational form of ethics consultation, ethical reflection is in fact induced by the presentation of a “case” to the respective ethics committee or by the organization of a case discussion on the ward. Thus, ethics comes into play at a point where there is a moral dilemma, e.g. in situations at the end of life when difficult decisions have to be made. This first implies that ethics is reduced to *moral* issues, which means that the ethical view as a reflection on “the good life” is not taken into account. Second, ethical reflection is primarily focused on the more or less momentary situation of *decision-making*.

### Participation of people concerned

An important question is whether the people concerned (patients, residents, relatives …) are sufficiently integrated into clinical ethics consultations (Fournier et al. 2009). The Nijmegen model refers—like many others (e.g., Baumann-Hölzle 2009; Stolper et al. 2016)—to a “multidisciplinary team” of health care professionals. However, even if, for example, relatives participate in a case conference, this does not necessarily mean that “well-practiced” hierarchical asymmetries do not give more weight to the perspective of doctors and nurses (especially if the “case” is presented in medical language).

### The unclear role of feelings

The principle-based clinical case discussion implies an analytical logic, in which (medical etc.) data and their possible evaluations are the center of interest. It requires an objectivizing distance. In German speaking countries, for example, virtually all instruments for ethical case discussions do not allow the consideration of feelings (Weidemann-Zaft and Schochow 2012). On the other hand, the importance of feelings in moral life is widely acknowledged and recently developed models of “Moral Case Deliberation” in the Netherlands take the crucial role of emotions into account (Molewijk et al. 2011).

### Transferring clinical ethics consultation to the nursing home?

Concerning the transfer of ethics from the hospital to the nursing home, it is advisable to consider the major differences in settings (Bockenheimer-Lucius 2012, 51). Whereas the orientational difference in hospital is health versus disease, the organizational aim in the nursing home lies in structuring everyday life, respecting the quality of life until the last day. The orientational difference here would consequently be life versus death, or life in dignity versus humiliation (Margalit 1996). In the nursing home, care and the organization of everyday life are more important than medicine—there usually is no medical personnel anyway. In medicine, decisions to be made normally concern treatment, which is rarely the case in nursing homes, where everyday ethical questions prevail. In reality, it is often not about “decisions”, but rather about arranging everyday life in a way that allows the person concerned to live in dignity (Schuchter and Heller 2012; Bollig et al. 2016). These comparative reflections alone show that it is not so simple to transfer clinical ethics consultation to the nursing home. Why should a model designed for medical treatment decisions be suitable for an environment in which treatment decisions are not part of the daily routine? The dominance and uncritical transfer of clinical ethics consultation to the nursing home would actually lead to a non-consideration of numerous ethical questions, as many points would simply be regarded as self-evident (cf. Schuchter and Heller 2016; Fiester 2015; Kohlen 2009).

### Focus on decision-making and/or on learning?

Apart from the principle-based case discussion, which—from an organizational point of view—is the most “successful” model of ethical reflection, also retrospective-oriented methods such as the “Hermeneutic Dialog” and the “Socratic Dialog” are described (Steinkamp and Gordijn 2003 and 2010, 280 et seq.). The first can help to carve out moral intuitions which are not immediately recognizable in a narrative, but hidden on a deeper level. The latter serves the clarification of basic concepts. On an organizational level, these methods are rather designed as learning arrangements for teams, the ethics committee or training. The gap between theory and immediate practical life is therefore wider. Orientation towards understanding and learning is more important than orientation towards a “solution” in a concrete situation where a decision has to be made. As a consequence, hermeneutic and Socratic reflection are rather marginal phenomena, both in discourse and in practice (exceptions and mixed forms are of course not excluded, cf. ibid. and Cadoré 1997).

There are two traditions which allow critical reflection. Both consider the concept of “care” as crucial:

1. The ancient ethics of the art of living (“care of the self”)
2. Care ethics (care for others)

## Care of the self: questions of “the good life” and the philosophical art of living

The reduction of ethics to moral issues was recently described by Roland Kipke as generally valid for bioethics (Kipke 2013). Ethics would therefore be a reflection on the justification and application of moral norms. Taking a look at the history of philosophy, however, one discovers that this focus on morals comprises only half of the ethical scope. The remaining half is what we call “the good life” or “happiness” (ibid.). For the respective disciplines of this ethical scope, various terms such as “art of living” and “ethics for thriving for the good life” are then used. Some oppose ethics as a theoretical reflection on the “focus on good life” to moral theory—a fact that does not reduce existing confusion. When the focus lies on Aristotle, terms such as “teleological” or “eudemonic” ethics are commonly used. When Hellenistic traditions serve as a reference (Stoicism, Epicurus), the terms “art of living” (Sellars 2009), “philosophy as a way of life” (Hadot 2005) and “care of the self” (Foucault 2009) are common.

The “teleological” perspective may of course be considered in ethics consultation, but only for the justification of moral judgments, as a moral principle to be referred to, so that actions can be judged (cf. Wils and Baumann-Hölzle 2013). Thus, as soon as some art of living comes into play, it is immediately subjected to remoralization. However, what is at stake is the far-reaching insight that art-of-living ethics has an entirely different inner logic (cf. Krämer 1995; Höffe 2009).

### Ethics beyond moral emergency

In this tradition, ethics is in fact not confronted with moral emergencies, but with everyday questions of the good life and an in-depth reflection of life as a whole (Ricoeur 2005, Chap. 5–7). Ethical issues are broader and as well more rooted in everyday life than medical-ethical emergencies. They concern the success or failure of life plans, self-respect, participation in the institutions of a community, and the significance or insignificance of our actions and experiences. They refer to the nature of love and the role of friendship in an accomplished life. In the end-of-life context, with its unavoidable experience of human suffering and the insight of the fragility and finite nature of life, we can say that ethics—in view of vulnerability, pain, suffering, fear of death and despair—struggles for an attitude, for wisdom and the ability to live it (cf. Hadot 2005; Schuchter 2016).

From this point of view, dilemma ethics in clinical ethics consultation concentrates too much on the isolated question what could need to be done in the concrete situation—as if it were an express repair service (cf. Appiah 2009, 203). Furthermore, ethics is not only about single actions, but especially about the actors themselves (cf. Frank 2004), who—in one or the other way—“practice” for life and create their self-image and picture of man in general.

### The Aristotelian and the Hellenistic paradigm of art-of-living ethics

Within the “ethics for thriving for the good life”, we can see a relevant difference in style, which has been rarely observed and not operationalized for practical life. In the history of philosophy, art-of-living ethics appear in two forms. On the one side, there is the Aristotelian paradigm, which is not too far away “from customs in modern universities” (Nussbaum 1996, 56); on the other side, we see the Hellenistic paradigm (Stoicism, Epicurus etc. following Socrates). What is remarkable is that, despite the works published by Pierre Hadot (2005), Michel Foucault (2009), John Sellars (2009) and—with restrictions—also Martha Nussbaum (1996), the Hellenistic art of living has not yet made it to the “textbooks” of (medical) ethics, which are largely dominated by Aristotelian thinking patterns.

In the Aristotelian art-of-living ethics, the main point is to acquire “outlines” (Höffe 2009, 98 et seq.). The tasks and outcome of philosophical reflection consist in developing the basic concepts of practical philosophy, such as happiness, virtue, action etc. In this way, an outline of the highest good (Aristotle NE1094a), a structural grid, so to say, the eudemonic skeleton (Höffe 2009, 98) of the good life is created. The acting person receives general guidelines and can then proceed to the decisive work, the accomplishment, which as such is to be seen as a non-philosophical process.

The art of living in the Hellenistic paradigm is fundamentally different. The crucial difference is that philosophy is seen as an exercise as such, and is an integral part of everyday life. Philosophy is not conceived as something first theoretical and then practical. It is not developed in seminars or on a piece of paper, i.e. in the procedural and organizational forms of mere theory or a primarily theoretical concept of philosophy. It is therefore seen as a process which takes place in practical life from the very beginning. As a consequence, it delivers less positive answers, but rather an opportunity to examine and question one’s own experiences in life (Schuchter 2016).

### Feelings as a major source of insight

Major emphasis is laid on feelings. In the Stoic tradition, feelings are not the opposite of reason—contrary to a rigidified prejudice. They are no irrational upheavals to be banned from a person’s thinking. The opposite is true: Feelings let us see the world in a certain light, and they include judgements. They are a major source of insight—and it is important to cultivate such emotions of ours, which allow us to get a reasonable and true picture of the world (cf. Nussbaum 1996).

### The Socratic way of thinking

The paradigm for such philosophic self-improvement is of course the conversation which Socrates initiated and conducted with his fellow citizens in the marketplace. The basic idea of Socratic conversation practice is that only through awareness and critical examination (“The unexamined life is not worth living”) of our unconscious and semiconscious existential convictions, which are deeply entrenched in our feelings and behavioral patterns, we can reach a point where we really understand and live our life. Such maieutic practicing in life demands repetition. In the *Seventh Letter*, Plato gets to the core logic of such philosophical ethics, when he says that the substantial insights are gained from permanent joint efforts for the cause in shared life (Plato, *Seventh Letter*, 341 c–d). In his *Letter to a Young Poet*, Rilke (cf. Rilke 1950, 21) writes that you have to love the questions in order to gradually live along some distant day into the answer. There is no shorter and at the same time more complete explanation for this Socratic philosophical movement of thinking.

In the project “Ethics from the bottom up” (see below), we made an effort to integrate the tradition of philosophical art of life (“care of the self”) and to “operationalize” it for organizational forms of ethics consultation.

## Care for others: care ethics

Another starting point for “Ethics from the bottom up” is care ethics. Care ethics was originally developed by Carol Gilligan (1991), when she analyzed Lawrence Kohlberg’s developmental psychological studies. The chief finding of Gilligan’s overall studies was the distinction of “the other voice” in moral reasoning, which could not be found in Kohlberg’s propositions, as they were marked by classical ethics derived from Kant’s theories.

Where a classical “ethics of justice” approach perceives values, norms and specific rules, a “care ethics” approach sees relationships and stories which people are involved in and which lead to concernment and compassion. The “solution” to a moral problem does not lie in judging on actions on the basis of moral principles, but in intensifying relationships and enhancing empathetic involvement. This allows not only to (a) find new options, which were not clearly discernible before, but also (b) to see and interpret a problem in a new, more plural way. In this approach, committed care would allow and favor “ethical creativity” and “practical wisdom” (Ricoeur 2005; Conradi 2001). As illustrated in the well-known Heinz Dilemma—through the lens of care ethics—the description of a morally challenging situation as a dilemma is not taken for granted. From the affected person’s view, single decisions are part of a whole story (cf. Frank 2004a). The (theoretical and practical) construction of a “moral dilemma” is therefore always an “abstraction” in the original meaning of the word, an “extraction” from the fluid context of life, from the previous and also the subsequent story. For this reason, care ethical thinking often goes hand in hand with narrative approaches in ethics (consultation) (Frank 2014).

## Paradigmatic accents of the Care Dialog

In the concept of the Care Dialog, a practical link between the two traditions described is established: care ethics and ancient art-of-living ethics (“care of the self”). Both care traditions share the same experience that ethical issues cannot be handled deductively by applying concrete and prefabricated norms, but only inductively in social processes, which respect the multidimensionality of problems and the singularity of human destiny (cf. Berger 2010, 604). Apart from this, both care traditions share the conviction that feelings play a crucial role when insights are gained and that philosophical work does change humans in some way or the other. Personal views are altered during the Care Dialog, as understanding and participation in the other person’s life are enhanced. Both ethical traditions do not give priority to rules for action, but to the acquirement of “practical wisdom”, which is a social and personal process. As Paul Ricoeur puts it, practical wisdom consists in developing behaviors that respect exceptions to the maximum and break the rules as little as possible (cf. Ricoeur 2005, 325). When a Care Dialog is arranged, it is not intended to infringe norms, but they fade into the background. The focus is no longer placed on the rule, but on changing perspectives and in-depth reflection. The encounter of humans and their personal views generate something new, therefore, the existential-communicative encounter is substantially relevant for the Care Dialog (cf. Berger and Heintel 1998, Jaspers 1994).

Thus, ethics comprises two efforts or “arts”, which are closely related to each other and which are both derived from the two lines mentioned above: ethics as the art of living and ethics of care; the latter being the effort and the art of understanding a person in his or her reality of suffering, which means entering with others into compassionate and caring relations. The former is the effort or the art of searching for and perhaps discovering possibilities of a good life (or consolation in dying and in mourning) in suffering with and for others. From this point of view, treatment decisions are only one part—namely the case of ethical emergency—of a continuing process of reflection and dialog. Ethics is a learning process.

In summary, a change from expert-driven clinical ethics to Care Dialogs implies the following displacements and the respective “operationalization” of ethics of care:

1. Subjects of ethical deliberations are not just dilemma situations, but meaningful experiences and situations in general, which concern the fundamental questions of human life.
2. The first “basic action” of ethical deliberation is not a principle-based reflection on a “case” represented primarily in medical facts, but it rather involves thinking about everyday existential issues on the basis of paradigmatic stories.
3. The second “basic action” of ethical deliberation is connecting with other people by making an effort to understand and to feel with others, which implies creating a setting that allows participation in other people’s destiny and views beyond social roles.
4. The essential question is less how to balance obligations or norms, but how to trace paths to a good life in the midst of suffering with and for others.
5. The objective of ethical deliberation is not just a singular decision, but the sustainable cultivation of collective practical wisdom in a web of meaningful relationships.
6. Ethics should be organized on the basis of everyday ethical reflection, situated in everyday practice in organizations, communities and so on—and not just when a moral dilemma occurs.

The project “Ethics from the bottom up” shall serve as an example for this organizational work.

## The pilot project “Ethics from the bottom up”

The project “Ethics from the bottom up” was put into practice in cooperation with a network of nursing homes, hospice and elderly care services in a rural region in Germany (details in Schuchter and Heller 2015). The project followed a participatory research approach in palliative care (Hockley et al. 2013), the main feature of which is to co-create knowledge between researchers and participants. The goal of participatory research is not to accumulate ‘pure’ knowledge for the scientific community, but also to initiate dialogical social learning processes in an organization, a community or a network. In our understanding, this tradition of research is consistent with the concept of “empirical ethics as a dialogical practice” based on “responsive evaluation” by Widdershoven et al. (2009). Our research interventions established (a) dialogs among practitioners (Care Dialogs) as well as (b) dialogs with them (ongoing cyclical process of analyzing data from documentation and providing results).

In a first step, a steering committee was established, which consisted of the initiating institutions together with management personnel from different palliative and elderly care services and institutions. The balance between competition and cooperation was a challenge in certain respects, but according to the metaphor of a “common infection with the hospice virus”, a shared interest and philosophy or ethical vision has been expressed. In concrete terms, this common will and understanding should be translated into an offer of support for family carers and relatives of severely ill, old and dying persons.

As is typical for participatory research, the aim of the project was twofold. With regard to research, the project aimed at exploring the concept of Care Dialogs; with regard to local care practice, the project intended to create settings, where concerns and ethical questions of relatives can be articulated, shared, discussed, and which contribute to mutual understanding.

The heart of the project consisted of three workshops where volunteers were trained in facilitating Care Dialogs. Participants of the workshops were in the first instance volunteers from the hospice service, and staff members from nursing homes and from the different nursing services in the community. At the end of the project, a stable core group of more or less 20 facilitators had evolved.

After each workshop, the participants of the workshops organized, moderated and documented Care Dialogs in nursing homes and outpatient environments. Documentation was continuously analyzed and interpreted by the research team so that findings from the first phase of the project (Care Dialogs after the first workshop) formed the input and a starting point for the next phase (second workshop and subsequent Care Dialogs), and so on. By doing so, interpretation and data analysis by the research team were validated by practice partners when they could find themselves in the issues carved out.

The first form of Care Dialog (including a respective documentation sheet), which has been rehearsed in the first workshop, resorts to the basic ideas of narrative ethics (cf. Frank 1997; Ricoeur 2005) and aims at generating and imparting care stories, and at documenting emotions and thoughts involved. The following questions could be used as an impulse:

- Which story is remembered and should be shared with others? What was the astounding or moving feature of it?
- Which pictures, feelings and insights emerge? What is the underlying issue? (“This story is about…”)
- Which practical conclusions can be drawn from the stories and the insights in the dialog?

A second form of Care Dialog (second workshop) is more or less derived from the Socratic Dialog (cf. Birnbacher 1999; Weiss 2015, Knox and Svendsen 2015). In the Socratic Dialog issues which emerge from narratives are subjected to in-depth reflection.

The impulse questions remain elementary and simple:

- What kind of experience do I have with this issue? Also from other sectors of life: What can I transfer, what not?
- How can I adjust my life accordingly?

Within a year, facilitators in seven institutions (more inpatient than outpatient) organized and conducted 20 (rather narrative than deepening) Care Dialogs, which all in all had a good, well-balanced mix of participants (mainly relatives but also staff members and residents of nursing homes). Thus, more than 80 short care stories could be documented and numerous topics and insights could be identified. When the project ended, Care Dialogs became part of the everyday routine in five institutions.

In the third workshop, key stakeholders in the field of palliative and elderly care were invited to discuss in how far the insights, questions and concerns expressed in the Care Dialogs were relevant for creating a “caring community”.

### Example: a Care Dialog and its existential questions

An example of a care story could be the following:

Mother sits in a wheelchair and lives in a nursing home. Her personality has changed as a result of dementia. A determined, formerly good mother becomes a permanently dominant, verbally aggressive and mean person full of mistrust. The mother is unhappy at the nursing home, takes a taxi to find an apartment, longs for her former independence and withdraws major amounts of money from her bank account. The judge has refused to appoint a guardian twice.

Furthermore, the following feelings (and therefore existential topics) were documented and are of ethical-philosophical relevance:

The daughter feels helpless, hurt and powerless as well as guilty about her ‘shortcomings’. She longs for the recovery of her former good relationship and has a need for recognition and appreciation. Listeners recognize themselves in the story. It is almost impossible to bear and understand that parents can undergo such horrible changes when they grow old or become sick. Is it allowed to be angry at one’s parents?

When practical consequences were discussed—and in this case the eight stories showed significant similarities—the issue of boundaries repeatedly came up: recognizing one’s own limits in time, setting boundaries, reducing exaggerated, partly unjustified feelings of guilt and blocking “excessive verbal violence coming from the mother”.

Concerning “Socratic in-depth reflection”, for example, some groups took “guilt” as their topic and gathered (Greek: *logos—legein*: gathering, collecting) many relevant experiences and ideas. Many family carers feel guilty because they believe not to have done everything they could for their mother or father; or they feel guilty at the mere thought of transferring their mother or father to a nursing home. Feelings of guilt are a significant burden, and they are nebulous and unspoken sentiments. Through the (“Socratic”) Care Dialog it was possible to find relieving differentiations and insights. The Socratic Dialog is not a therapeutic dialog—participants are not addressed in their neediness and their deficits; quite the contrary: they offer and generate together life-serving knowledge on the basis of personal recollection and experience—in philosophical terms: practical wisdom is being cultivated. The participants enter into a hermeneutic process of collective self-enlightenment. It is an aha experience and “a difference that makes a difference” for one’s life to discover that the nebulous feeling of guilt does not have its origins in a particular decision or action, but, for example, in an overall life attitude, in the assumed expectations of others or—quite different—in one’s gratitude towards one’s parents. (More than 10 forms of “guilt” have been documented.)

Another group chose “the slowness of understanding in the experience of loss”. In this way, experiences remembered were analyzed and subsumed under certain terms.

### Chances and limits of “Ethics from the bottom up”

Where do we see the chances and benefit of Care Dialogs? We received feedback mainly from members of the steering committee, who also participated in the workshops and co-facilitated Care Dialogs.

Care Dialogs have

- a socially connecting potential, as they enhance the feelings of mutual concern and reassurance.
- They allow comprehension in a circular process, as the participants make an effort to understand and receive understanding in return.
- In the Care Dialog, the participants—e.g. relatives of older or dying people—are perceived and respected with their full life experience, knowledge and competence, and not only as persons involved, who need therapy, an easing of the burden or training. According to feedback we received from some participants, Care Dialogs are a success especially because their framework is not the one of a self-help group.
- Care Dialogs can be facilitated by everybody, there is no need for professionals and therefore they can always be initiated from the bottom up. The call for a supervisor, psychotherapist, medical ethicist or moderator—at least for difficult situations or issues—was clearly considered to be unnecessary at an evaluation steering committee meeting. Our observation on this issue is that self-reflection on existentially profound questions can be exercised by everyone,
  - when dialogs are embedded in an already existing and to some extent sustainable network of relationships (between members of staff, relatives, hospice volunteers …)
  - when a protective environment is created, and
  - when dialog partners “open up existentially” and therefore give others the chance to do the same—especially once it comes to analyzing and talking about feelings. For example, it was possible to talk about feelings of guilt, a fact that does not seem self-evident to us.
- Finally, ideas and solutions to specific problems could be developed. (One participant, for example, put a poster with the recommendations on a wall at home.)

Which limits did we perceive in the pilot project?

- First of all, the “Socratic in-depth dialog” is somewhat more demanding and requires permanent participation in the group, as otherwise people have to get to know each other through (time-consuming) story-telling.
- The dialogs were documented by the facilitators, but analyzed mainly by the university project partner. Many insights and findings can only be obtained by analyzing documentation. At this point, there is still potential in enabling institutions, networks and communities to do so. Without transmitting results (insights and crucial questions) from documentation to others, the hermeneutic social learning process will more or less remain limited to concrete participation in the dialog groups or to the varying ability of participants to communicate questions and knowledge in their organizations or families. When the project ended and Care Dialogs continued, facilitators ceased documenting. Care Dialogs have a great value as such, but the project failed (at least in a structured way) to transfer tacit experiential knowledge into more explicit ‘systemic’ knowledge (Willke 2004).
- Group discussions can be easily organized in nursing homes, in outpatient settings individual dialogs were usually arranged.
- In certain situations, it can be helpful to embed a “classical” but simple decision model in existential dialogs. One manager of a nursing home indicated that some participants would “need more structure” in the dialog.

## Discussion

The project “Ethics from the bottom up” is to be seen as a pilot project with limited resources. Evaluating feedback was received in the course of the project. For us, however, the implementation of the basic theoretical assumptions mentioned above reveals relevant insights and questions for the future development of ethics consultation in the healthcare system. We see the main contribution of our empirical study and our theoretical considerations in the following points:

- organizing deliberation on ethical issues beyond moral dilemmas
- encounter of persons with their stories
- consistent participation of people concerned (relatives, residents etc.)

### Ethical deliberation beyond moral dilemmas

Whereas classical (prospective and retrospective) moral case deliberation has a strong focus on moral issues when dilemma situations occur, Care Dialogs start from meaningful experiences and situations in general which concern the fundamental questions of (a good) life (despite suffering). Therefore, concept and practice of Care Dialogs are consistent with the logic of the art-of-living ethics in its Socratic-Hellenistic expression.

For this purpose, Care Dialogs lay a major emphasis on the expression and interpretation of feelings. Within a classical medical—ethical framework, the above-mentioned story could appear to contain a conflict between the principle of autonomy and the principle of beneficence, but when feelings are interpreted (cf. similar: Molewijk et al. 2011), other and deeper ethical issues, which are vital to the “good life despite suffering” of individuals concerned, become visible. Furthermore, these topics are not predefined by professional moderation or guidelines. They are identified by the affected individuals themselves as crucial existential issues: In the example above, this would be the existential experiences of powerlessness, guilt and yearning for good relationships, which demand maturing into an ethical position, as well as questions about understanding the (primarily) unintelligible, bearing the unbearable, identity, changes in personality and the legitimacy of anger, which Aristotle and Seneca already raised (cf. Nussbaum 1996). These are altogether profound, even abysmal philosophical-ethical issues, which cannot be quickly solved. Sometimes there is no definite solution at all. The ethical act is then to first identify these existential concerns and topics through the narrative dialog—an undertaking that is anything but trivial in everyday life—then share these stories with others and in this way, maybe and with no ultimate guarantee, find an answer and develop an existential position (ethopoiein).

### A narrative encounter: thinking and communicating with stories

Whereas in the standard model of moral case deliberation, but also in the Hermeneutic Dialog (Steinkamp and Gordijn 2003) and sometimes even in the (neo-)Socratic Dialog, one specific “case story” is analyzed (mostly in a detailed protocol like in Stolper et al. 2016), Care Dialogs follow a different approach: The “basic operation” of the dialog is not to analyze one story (singular), but to share several stories (plural) in order to find connections and differences, to identify crucial issues and to learn from different experiences.

Narrative ethics theory therefore makes a clear difference between thinking *about* stories and thinking *with* stories (Frank 1997; Morris 2001). The basic moral operation does not consist in a rational analysis of the story in search of moral judgements, values and problems contained—this would actually imply a reduction of the story to something else. The most important point is the impact of a story on the person. The primary aim is not to work on the story, but that the story works “on” and “in” the dialog partners (Morris 2001). This is the first ethical core operation in ethics of care: getting more deeply involved in other people’s history and therefore enlarging, changing and deepening perspectives. From Plato to Jaspers as well as in care ethics, the conviction prevails that an existentially deepening contact between people favors creativity and new ideas. As a result, ethics of care focuses more on the social arrangement and process of ethical-existential communication than on the refinement of the protocol (in the analysis of the judgement).

### From the bottom up: participation and the perspective of the persons concerned

Whereas in the clinical standard model “client participation” is not intended primarily or is perceived as “a precarious relational balance” (Weidema et al. 2011), Care Dialogs consistently realize participation of people concerned (relatives and residents). Even more, whereas usually individual clients or relatives seem to “participate”—if at all—in a group of professionals, Care Dialogs are originally designed for ethical deliberation among people concerned.

This implies the consistent consideration of the affected person’s view (and also the perspectives of relatives), as well as the search for a level of communication which does not put anyone in a superior or inferior position.

This is reflected in the narrative framework and structure of the dialogs. The structure of narratives and their framework have a decisive impact on the perception of the problem and the moral quality of relationships (Frank 1997; MacIntyre 1984). A clinical-ethical case discussion usually starts as follows: “Mr. S., 83 years, coronary heart disease, acute renal insufficiency …” etc. In contrast to this, the narratives of Care Dialogs begin like this: “My mother has always been a good person, but now …” In the first case, a primarily medical-therapeutic story is developed from information given by healthcare professionals. Its aim is to discern preferences concerning (non-)treatment. Therefore, biographic aspects are an episode of the medical story, as they supply information for decision-making. In the second case, we see the opposite approach: The first words already set the narrative framework of a personal history in which the medical-therapeutic story and the respective decisions are an episode. In the first case, the professionals’ perception of the problem shapes the consultation; in the second, it is the perception of the people concerned (cf. Frank 1997).

The only chance to equalize asymmetries and create understanding beyond social roles is to give priority to the elementary story-telling of individuals concerned. By giving narratives (stories) the status of the central language game in ethical deliberation, the dominance of expert knowledge is annulled in favor of a democratization of the opportunities to speak and a consequent participation (for instance, by relatives).

For professionals, this means that they are ready to consider and integrate feelings like vulnerability and insecurity (Weidema et al. 2011) instead of ignoring them. This kind of integration can be seen as the “mature” practice of medicine and care, which increases professionalism instead of relativizing it (cf. Frank 2004).

## Practical conclusion

The “Ethics from the bottom up” approach originarily refers to the communal space and the nursing home. This implies two aspects: (a) skepticism whether clinical ethics consultation should directly be applied to the nursing home and further to the community and (b) a critical view on clinical consultations practiced in hospitals. We can ask the opposite question: Which lessons from such originarily community or nursing home ethics can be integrated into clinical ethics consultation?

Our first suggestion is to organize settings of ethical-philosophical deliberation specifically designed for (consistent participation of) people concerned (relatives, patients, residents, clients etc.), and to ensure that insights, concerns and questions of such or similar Care Dialogs be transmitted systematically to health care professionals and have an impact on communication and practice in health care organizations.

Our second suggestion (extending the first suggestion) is to rethink and reorganize the two main directions of ethics consultation, the one focusing on decision-making, the other one on understanding and learning. The organization of ethics in health care organizations, networks and in communities should neither focus retrospectively on understanding nor prospectively on solutions, but it should pave the way for socially and existentially sustainable approaches to care situations. The organizational solution would actually be to embed ethical questioning in already existing communication formats (on the same issue: von der Dam et al. 2011), in order to increase awareness and wisdom in those existential issues which keep bothering patients and their relatives. Results and questions of the proposed form of Care Dialogs are, in our experience, sufficiently simple and substantial to do so. Thus, it would be possible during a ward round to talk not only about medical findings, but also—in a few sentences—about existential issues and the patient’s “spiritual” well-being. Ethical issues and insights could then be passed on at the shift-handover and later be discussed in organizational meetings with the management, where they could be linked with ethical questions on an organizational and business level. This approach would furthermore avoid the “delegation trap”—which means that ethics consultation is in fact passed on to the ethics committee—and avert the separation of clinical and organizational ethical questions. Through an intelligent connection of consultation settings, implying that information is passed on and feedback is given in both directions, ethics becomes systemically effective (Krobath and Heller 2010; Krainer and Heintel 2010). Thus, our hypothesis could be the following: Such “Ethics from the bottom up”, which is transferred from the community and nursing home to hospital, can contribute to the prevention of many (clinical and organizational-ethical) “dilemma cases”—first, through sensitivity and wisdom in recurring existential topics; second, by facilitating a social-communicative process within organizational routines. Even if there is still the need for a standard case conference (on a clinical or “business” level), its resources in terms of shared knowledge and the social context are much better than if it just “breaks in”. In short: Care Dialogs create collective practical wisdom (cf. Weick and Roberts 1993).

Therefore, it makes sense—and this is our third suggestion—to design a case conference in a way which integrates as many ideas of Care Dialogs or as many aspects of (both traditions of) ethics of care as possible, such as: the reflection of feelings, deriving values and norms from lived experience and not from moral theory; focus not only on decision-making, but also on collective learning and mutual understanding. This seems to be the case in the concept and practice of “Moral Case Deliberation” (MCD), as developed in a series of projects in the Netherlands (Molewijk et al. 2008; Stolper et al. 2016).

## Perspective

In the theoretical discussion of “ethics of care” and the respective empirical experiences, we could depict that current debates in medical ethics and ethics consultation show some reductionism. Apart from this, we could also generate hypotheses for the further development of ethics consultation in the healthcare system.

With a glimpse to the future and to current developments, we would like to reflect briefly upon the potential of Care Dialogs from a public health point of view on end-of-life care. This includes preventive aspects, like in Advance Care Planning (ACP), and political aspects as reflected in “health promoting palliative care” or the “compassionate community” movement (Kellehear 1999, 2012; Wegleitner et al. 2015).

As to the first point, the “caring” and “preventive” aspects of Advance Care Planning and Care Dialogs given, we would like to highlight important differences between ACP and Care Dialogs. This does not mean that Care Dialogs could replace ACP—which is not the case—but we would like to indicate why ACP initiatives—as currently implemented in Germany (in der Schmitten et al. 2014)—should be complemented by Care Dialogs (or similar reflective practices).

ACP aims at “respecting choices” concerning medical treatment at the end of life and, by this, at preventing from possible harm of medical (over-)treatment. Conversation processes, in one way or the other, aim at the completion of a written advance directive. On the other hand, Care Dialogs aim at preventing from the most negative consequences of existential suffering in general, and conversation processes are an open inquiry into fundamental “philosophical” questions and concerns when faced with borderline situations. Furthermore, ACP creates a (formal) network between professional institutions in order to guarantee that advance directives are respected; Care Dialogs, in addition, have the potential to create a web of caring relationships among people concerned and between the formal and informal care system. They contribute to building a caring community through shared stories of care (Schuchter and Heller 2015). In all these respects, Care Dialogs are suited to open up a narrowing focus on medical treatment in favor of a public health approach, when people try to get along with severe illness, old age, dying and mourning (see also Borgstrom and Walter 2015).

In this sense and as regards the second point, the political philosopher and care ethicist Joan Tronto (2013) suggests that a caring society and a caring democracy require settings where people can learn from and about the lives of others. This is the potential of Care Dialogs. Therefore, the ethical and social scope of Care Dialogs is broader, and it is not limited to “optimizing” medical treatment decisions in unclear situations. It does have a political, even “enlightening” role, as it democratizes ethics itself, and ultimately empowers citizens to develop positions and their own language for crucial questions of living and dying.
